# Cisplatin-Loaded Thermosensitive Liposomes Functionalized with Hyaluronic Acid: Cytotoxicity and In Vivo Acute Toxicity Evaluation

**DOI:** 10.3390/pharmaceutics15020583

**Published:** 2023-02-09

**Authors:** Isabela Pereira Gomes, Juliana de Oliveira Silva, Geovanni Dantas Cassali, André Luís Branco De Barros, Elaine Amaral Leite

**Affiliations:** 1 Department of Pharmaceutical Products, Faculty of Pharmacy, Federal University of Minas Gerais, Av. Antônio Carlos, 6627, Belo Horizonte 31270-901, MG, Brazil; 2 Department of General Pathology, Institute of Biological Sciences, Federal University of Minas Gerais, Av. Antônio Carlos, 6627, Belo Horizonte 31270-901, MG, Brazil; 3 Department of Clinical and Toxicological Analyses, Faculty of Pharmacy, Federal University of Minas Gerais, Av. Antônio Carlos, 6627, Belo Horizonte 31270-901, MG, Brazil

**Keywords:** multi-functionalized liposomes, antitumor drug, toxicity, breast cancer, hyperthermia

## Abstract

Cisplatin (CDDP) is a potent antitumor drug used in first-line chemotherapy against several solid tumors, including breast cancer. However, toxicities and drug resistance limit its clinical application. Thermosensitive liposome (TSL) functionalized with hyaluronic acid (HA) containing cisplatin (TSL-CDDP-HA) was developed by our research group aiming to promote the release of CDDP in the tumor region under hyperthermia conditions, as well as to decrease toxicity. Thus, this study aimed to evaluate this new formulation (HA-coated TSL-CDDP) concerning in vitro behavior and in vivo toxicity compared to non-coated TSL-CDDP and free CDDP. Cytotoxicity assays and nuclear morphology were carried out against triple-negative breast cancer cells (MDA-MB-231), while an in vivo toxicity study was performed using healthy Swiss mice. The results showed an increase (around 3-fold) in cytotoxicity of the cationic formulation (non-coated TSL-CDDP) compared to free CDDP. On the other hand, TSL-CDDP treatment induced the appearance of 2.5-fold more senescent cells with alteration of nuclear morphology than the free drug after hyperthermia condition. Furthermore, the association of liposomal formulations treatment with hyperthermia increased the percentage of apoptotic cells compared to those without heating. The percentage of apoptotic cells was 1.7-fold higher for TSL-CDDP-HA than for TSL-CDDP. For the in vivo toxicity data, the TSL-CDDP treatment was also toxic to healthy cells, inducing nephrotoxicity with a significant increase in urea levels compared to the saline control group (73.1 ± 2.4 vs. 49.2 ± 2.8 mg/mL). On the other hand, the HA-coated TSL-CDDP eliminated the damages related to the use of CDDP since the animals did not show changes in hematological and biochemical examinations and histological analyses. Thus, data suggest that this new formulation is a potential candidate for the intravenous therapy of solid tumors.

## 1. Introduction

Cisplatin (cis-diaminodichloro-platinum (II), CDDP) is an anticancer drug used in many solid tumors’ treatment, including those of the head, neck, lung, testis, ovary and breast. It is a highly effective chemotherapeutic agent, but it has many toxic effects such as severe nephrotoxicity, myelosuppression, ototoxicity] and neurotoxicity. These can even lead to the interruption of treatment by the patient [1]. However, the major problem related to its use is nephrotoxicity, typically characterized by acute kidney injury, which occurs in 20–30% of patients. Different strategies have been studied for decades in order to reduce its toxicity and improve its antitumor efficacy [2].

An alternative widely discussed is the use of CDDP-loaded liposomes, which act as modulators of pharmacokinetics and pharmacodynamics. The lipid composition of these nanocarriers can change drug half-life, biodistribution and permeability, in addition to allowing tumor targeting, consequently reducing toxicity [3]. Liposomal formulations carrying CDDP (SPI-77, Lipoplatin, and LiPlaCis) have been investigated in advanced clinical phase studies for non-small cell lung, breast and pancreatic cancer treatment. Although these formulations have considerably reduced the adverse effects associated with CDDP, slow or inefficient drug release is still a problem that might result in lower efficacy [4,5]. In this sense, different strategies to circumvent these problems have been evaluated and thermosensitive liposomes (TSL) bring some advantages, as they are designed to deliver the drug specifically after moderate heating in the tumor region (hyperthermia). They are prepared from phospholipids, such as 1,2-dipalmitoyl-sn-glycero-3-phosphatidylcholine (DPPC), that exhibit a sharp gel-to-liquid crystalline transition at mild temperatures. In the gel phase, the ordered chains allow the nanostructures’ formation, while in the liquid crystal phase a gradual increase can be observed in the mobility of the molecules in the bilayer which becomes more disordered and favors the drug release [6,7,8].

Another well-defined strategy for selectively killing tumor cells is to decorate the liposome surface with ligands whose receptors are overexpressed in these cells. CD44 is a transmembrane cell protein highly expressed in various cancer cells with a strong affinity for hyaluronic acid (HA), a natural, non-toxic, biodegradable and non-ionic polymer [9,10,11]. HA has been used to coat cationic liposomes via electrostatic interaction, or it can be covalently coupled to the polar headgroup of the lipid [10,11,12,13]. In addition, it might contribute to reducing the adsorption of blood proteins as well as increasing cell uptake by endocytosis [14,15].

Based on this, our group recently developed, CDDP loaded-thermosensitive liposomes (TSL-CDDP) coated with HA by electrostatic interaction, (TSL-CDDP-HA) for active targeting. The formulations were composed of DPPC, cholesterol (CHOL), and stearyl-amine (SA). Phase transition temperature was similar for HA-coated and non-coated TSL formulations. The thermo-sensitivity of the system was clearly evidenced by changes in the supramolecular organization due to the local misorientation in the lipids’ structure, confirming the conformational alteration due to warming [16].

Considering possible clinical applications of these thermosensitive nano-systems for cancer treatment, it becomes mandatory to evaluate their pre-clinical toxicity, since an effective translation can only be processed after guaranteeing the safety profile of the formulation. Therefore, in vitro studies of cytotoxicity, and acute toxicity after intravenous (IV) administration in healthy Swiss mice were carried out.

## 2. Materials and Methods

### 2.1. Materials

Hyaluronic acid (HA) 4 kDa was supplied by Innovasell (São Paulo, Brazil) and 1,2-dipalmitoyl-sn-glycero-3-phosphatidylcholine (DPPC) was purchased from Lipoid GmbH (Ludwigshafen, Germany). Dulbecco’s Modified Eagle’s Medium (DMEM), fetal bovine serum, penicillin/streptomycin, sulforhodamine B, cholesterol (CHOL), and stearyl-amine (SA) were purchased from Sigma Chemical Company (St. Louis, MO, USA). Cisplatin was acquired from Quiral Química do Brasil (Juiz de Fora, Brazil). Polycarbonate membranes were purchased from Millipore (Billerica, MA, USA). Sodium diethyldithiocarbamate (DDTC) was supplied by Sigma-Aldrich Company (St. Louis, MO, USA). The water was purified on a Milli-Q^®^ Direct-Q3 Millipore system (Billerica, MA, USA). The human breast adenocarcinoma cell line MDA-MB-231 was purchased from the American Type Culture Collection (Manassas, VA, USA). The mice used in the study were obtained from the vivarium of the Faculty of Pharmacy-UFMG (Belo Horizonte, Brazil). Biochemical analysis kits were obtained from Labtest (Lagoa Santa, Brazil) and Bioclin (Belo Horizonte, Brazil). All other chemicals were analytical or HPLC grade and were used without further purification.

### 2.2. Preparation of Cisplatin Thermosensitive Liposomes (TSL-CDDP)

The thermosensitive formulations were prepared by the reverse phase evaporation method [16]. Liposomes were composed of DPPC:CHOL:SA in lipid molar ratios of 95:2.5:2.5 for a total lipid concentration of 40 mmol L-1. For the preparation of CDDP-loaded liposomes (TSL-CDDP), CDDP solution (2.0 mg/mL) was prepared in 0.9% (*w*/*v*) NaCl and added to the lipid film dissolved in ethyl ether. After size calibration, the untrapped CDDP was separated from the liposomes by ultracentrifugation (Optima^®^ L-80XP ultracentrifuge, Beckman Coulter, Brea, CA, USA) at 44,800× *g* at 10 °C for 30 min. The pellet was reconstituted in 0.9% (*w*/*v*) NaCl to obtain the initial volume of the liposomal formulation. TSL and TSL-CDDP were functionalized with 4 kDa HA by electrostatic interaction according to the method described by Ravar and collaborators [17].

### 2.3. Determination of Diameter and Zeta Potential

The mean diameter and polydispersity index (PDI) of the vesicles were determined by Dynamic Light Scattering (DLS) at 25 °C using a fixed angle of 90° (Zetasizer Nano ZS 90, Malvern Instruments, Malvern, UK). The samples were diluted 40 times in 0.9% (*w/v*) NaCl solution previously filtered (0.45 µm filter, Millipore).

The zeta potential was determined by DLS associated with the electrophoretic mobility of the vesicles at a temperature of 25 °C using a fixed angle of 90° (Zetasizer Nano ZS 90 equipment, Malvern Instruments, Malvern, UK). Samples were diluted 40-fold in filtered 1 mM NaCl (0.45 µm filter, Millipore). Zeta potential measurement was used to evaluate the efficiency of HA coating on TSL-CDDP by electrostatic interaction since a neutralization in the surface charge might indicate the association of HA to the liposome surface. All results were expressed as the mean ± standard error of three lots of each formulation.

### 2.4. Content of Cisplatin Encapsulation

The encapsulation percentage (EP) of CDDP in the liposomal formulations was determined by high-performance liquid chromatography (HPLC), using a derivatization method with sodium diethyldithiocarbamate (DDTC) previously reported by Gomes et al. [16]. Briefly, EP was based on determining the concentration of CDDP in the liposomes before (unpurified liposomes) and after centrifugation (purified liposomes). To an aliquot of liposome (250 µL), acetonitrile (500 µL) was added followed by vortexing for 1 min and centrifugation at 10,000 rpm for 10 min. The supernatant (250 µL) was transferred to an Eppendorf and dried under an N2 atmosphere. The residual product was resuspended in 1 mL of 0.9% (*w*/*v*) NaCl, and then 250 µL was used to react with a solution of DDTC in NaOH (600 µL of a 1 mM solution). The samples were incubated at 37 °C for 60 min and extracted with 250 μL of chloroform. After separating the layers, the chloroform layer was filtered through a 0.45 µm filter and injected into the chromatographic system [16]. The chromatographic apparatus of the HPLC analysis consisted of a quaternary pump (G1311B), an auto-injector (G1329B) and a diode array detector (DAD) (G4212B) connected to the EzChrom integration program (Agilent Technologies, Santa Clara, CA, USA). Separation was performed using a 250 cm × 25 mm with particle of 5 μm Hypersil C18 column (Agilent Technologies, Santa Clara, CA, USA). The mobile phase composed of methanol and water (65:35 *v*/*v*) was filtered and degassed by suction-filtration through a nylon membrane. The flow rate was 1.5 mL min^−1^ in isocratic flow, and the injection volume was 20 μL. The eluate absorbance was monitored at 254 nm [16].

### 2.5. In Vitro Experimentation

Human breast tumor cells (MDA-MB-231, ATCC HTB-26) were cultured in DMEM culture medium, supplemented with FBS (10% *v*/*v*), penicillin (1% *w*/*v*) and streptomycin (1% *v*/*v*), and maintained in a humidified incubator containing 5% CO_2_ at 37 °C. Upon reaching the confluence stage, the cells were trypsinized, and an aliquot was transferred to another flask containing a complete culture medium for subculture.

#### 2.5.1. In Vitro Cell Viability

MDA-MB-231 cells were plated in 96-well plates at 1 × 10^4^ cells/well (*n* = 3). The plates were pre-incubated for 24 h in a 5% CO_2_ atmosphere at 37 °C. Subsequently, the cells were treated with TSL-CDDP, TSL-CDDP-HA and free CDDP at concentrations of 12.5; 25; 50, and 100 µM and the respective blank formulations. This experiment was conducted with and without hyperthermia for comparison purposes. Thus, the plates without hyperthermia were kept for 48 h at 37 °C. For the hyperthermia approach, the plates were kept for 6 h at 37 °C to permit drug internalization, followed by 1 h for hyperthermia at 42 °C [18,19] and then 41 h at 37 °C to complete the same 48 h used for the group with no heating. For 1 h heating, a thermo-shaker (KS 4000 I control, IKA, Wilmington, NC, USA) at 50 rpm was used. Brightfield microscopy images were taken after heating and after 48 h of incubation, and cell viability was then assessed by sulforhodamine B assay and reading performed on an Elisa VersaMax™ reader (Molecular Devices, LLC, San Jose, CA, USA). IC50 was obtained by non-linear regression analysis using GraphPad Prism^®^ Version 6.0 software (GraphPad Software Inc., San Diego, CA, USA).

#### 2.5.2. Nuclear Morphology Analysis

MDA-MB-231 cells were plated in 6-well plates at 1 × 10^4^ cells/well. The plates were pre-incubated for 24 h in a 5% CO_2_ atmosphere at 37 °C. Subsequently, the cells were treated with TSL-CDDP, TSL-CDDP-HA, and free CDDP at the IC50 of each formulation. Again, the experiment was conducted with and without hyperthermia for comparison purposes using the same protocol described above. After 48 h of treatment, cells were fixed with 4% formaldehyde for 10 min and then stained with Hoescht 33,342 solution (0.2 µg/mL) for 10 min at room temperature in the dark. Fluorescence images of the nuclei were obtained using an AxioVert 25 microscope with a Fluo HBO 50 fluorescence module connected to the Axio Cam MRC camera (Zeiss, Oberkochen, Germany). The cores were analyzed using the Image J 1.50i Software (National Institutes of Health, Bethesda, MD, USA, 2016) and the “NII_Plugin” plugin available at http://www.ufrgs.br/labsinal/NMA/ (accessed on 4 November 2020).

### 2.6. In Vivo Toxicity Study

Female Swiss mice, 6 weeks old, weighing 25 ± 1.5 g, were obtained from the Faculty of Pharmacy, Federal University of Minas Gerais. The animals were housed in plastic cages with free access to food and water and kept in an area with a light/dark cycle control. All protocols were approved by the Ethics Committee on the Use of Animals of the Federal University of Minas Gerais (Protocol #218/2020, approval date 9 November 2020). The animals were separated into four groups with six animals per group, and they received intravenous treatments with TSL-CDDP, TSL-CDDP-HA, free CDDP, or NaCl 0.9% (*w*/*v*) as a control group. The administered dose was 10 mg/kg, chosen based on a previous study carried out by our research group, in which the maximum tolerated dose (DMT) for female mice treated with free CDDP was close to 10 mg/kg [20]. For 14 days, the behavior and weight changes, besides signs of toxicity or death, were monitored. At the end of the experimental period, the mice were anesthetized with a mixture of xylazine (15 mg/kg) and ketamine (80 mg/kg) and blood samples were collected from the brachial plexus and transferred to tubes containing 0.05 mL of EDTA 18% (*w*/*v*) for hematological and biochemical examinations. Serum was obtained by centrifuging blood at 5000 rpm for 6 min. The hematological parameters evaluated included red blood cell (RBC) count, hemoglobin, hematocrit, white blood cell (WBC) and platelet count. All analyses were determined using an automated analyzer (Hemovet^®^ 2300 São Paulo, Brazil). Alanine aminotransferase (ALT) and aspartate aminotransferase (AST) were assessed as markers of liver function, and urea and creatinine as renal function. All biochemical assays were performed with a semiautomatic analyzer model Bio plus BIO-2000 (São Paulo, Brazil) using assay kits from Labtest Diagnóstica (Lagoa Santa, Minas Gerais, Brazil).

For histopathological analysis, kidneys, liver, spleen, intestine and bone marrow were removed and fixed in 10% (*v*/*v*) buffered formalin. Tissue fragments were embedded in paraffin blocks, sectioned into 5 µm thick sections and stained by the hematoxylin-eosin method. The images were obtained using an optical microscope connected to a camera.

### 2.7. Statistical Analysis

The analyses of normality and homogeneity of variance were performed using the D’Agostino and Pearson, and Bartlett tests. Variables that did not follow a normal distribution were transformed by the log (variable). Data from liposome characterization, nuclear morphology and in vivo studies were tested using one-way ANOVA, followed by the Tukey test. In vitro cytotoxicity data were tested by two-way analysis of variance. Differences were considered significant when the *p*-value was less than 0.05. The program used for all statistical analysis was GraphPad Prism 6.0.

## 3. Results

### 3.1. Average Diameter, PDI, Zeta Potential and EP

The results of liposomes’ characterization are summarized in Table 1. The mean diameter and polydispersity index indicate uniformly distributed vesicles in a small size range. Regarding the HA coating, the zeta potential close to neutrality confirms the success of the liposome surface coating since the coating strategy is based on charge interaction. The percentage of CDDP encapsulation in TSL was lower than that found by Leite and coworkers [20], probably due to the membrane rigidity of thermosensitive liposomes, composed of rigid lipids [7]. It is important to mention that even with a low drug loading the amount of CDDP in liposomes was still suitable for further in vitro and in vivo studies.

### 3.2. In Vitro Cell Viability

**Figure 1:**
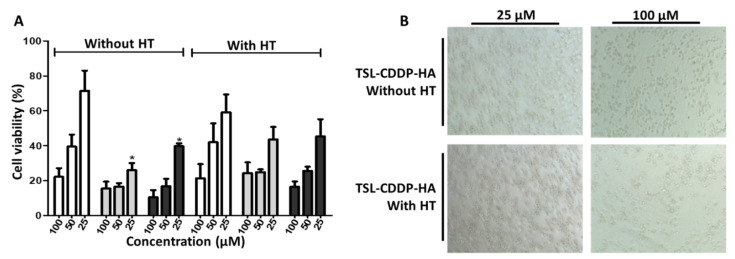
(**A**) Cell viability of MDA-MB-231 after treatment with free CDDP (white bars), TSL-CDDP (gray bars), and TSL-CDDP-HA (black bars) formulations without and with HT, at concentrations of 25, 50, and 100 µM. (**B**) Brightfield microscopy images, 20× objective, after 48 h of treatment with the formulation TSL-CDDP-HA, with and without HT. Data are expressed as mean ± error of three independent experiments. * Represents a significant difference when compared to free CDDP (*p* < 0.01, Bonferroni’s test).

The cytotoxicity of drug-containing formulations, as well as the free drug, was evaluated by the sulforhodamine B method. The cell line MDA-MB-231, a triple-negative breast cancer cell (TNBC), was selected as an in vitro model due to its high expression of CD44 receptors (>30%), which are receptors for HA [21]. For CDDP-treated groups, a dose-dependent reduction in cell viability was observed (Figure 1). Considering the treatment with the TSL-CDDP without hyperthermia, it was possible to verify that the cell viability was significantly reduced compared to the free CDDP (*p* < 0.01) at a concentration of 25 µM. The IC50 for this formulation was 16.6 µM, demonstrating greater cytotoxic effectiveness compared to the free CDDP (50.3 µM), as shown in Table 2. On the other hand, the formulations containing HA (TSL-CDDP-HA) showed no significant difference compared to the free drug.

Furthermore, it can be observed that there was no difference in cytotoxicity between the formulations with and without HT, probably due to the heating protocol used. Alavizadeh and coworkers applied a different hyperthermia protocol than the one developed in the present study, in which cells were heated for 1 h at 37 or 42 °C in a complete medium and then incubated for only 3 h at 37 °C in a CO_2_ incubator. The plates were then washed and replaced with a liposome-free medium and incubated for an additional 48 h at 37 °C [19]. This replacement of the medium could prevent cell death evenly in the plates with and without hyperthermia, thus allowing better visualization of the results. In the brightfield microscopy images after 48 h of treatment (Figure 1B) it was observed that, only for the TSL-CDDP-HA with a dose of 100 μM, it was possible to visualize lower cell viability with the use of hyperthermia. In these images, changes in cell morphology were also observed after treatment with TSL-CDDP-HA at higher concentrations, with the presence of nuclear condensation indicating the possible occurrence of apoptosis.

### 3.3. Analysis of Nuclear Morphology

The nuclear morphology analysis proposed by Filippi-Chiela [22] uses the nuclear morphometric analysis tool (NMA), and the data are generated in the Image J software. This tool classifies and distributes the nuclei in normal (N), irregular (I, mitotic catastrophe), small regular (SR, apoptosis), small (S, mitosis), small irregular (SI, mitosis with damage or nuclear fragments), large regular (LR, senescence) and large irregular (LI, mitotic catastrophe) [22].

**Figure 2:**
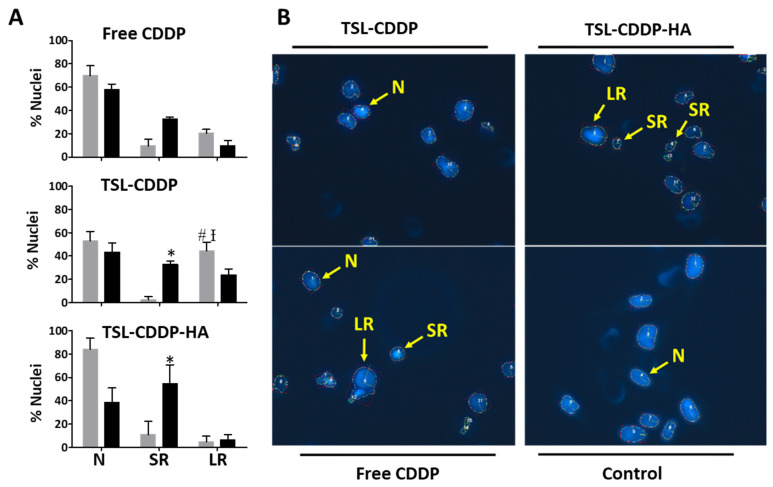
(**A**) Nuclear morphometric distribution of MDA-MB-231 cell line exposed to IC50 of different treatments for 48 h, without (gray bars) or with hyperthermia (black bars). (**B**) Fluorescence images of nuclei from the cell line MDA-MB-231, stained with Hoescht, obtained after 48 h of the different treatments at the IC50 of each formulation. Enhancement, 40×. Data are expressed as mean ± error standard of three independent experiments * Significant difference between heating and no heating for the same group of treatment. # Significant difference from free CDDP. Ɨ Difference from TSL-CDDP-HA. *p* < 0.05 (Tukey’s test). Abbreviations: N: normal; SR: small and regular; LR: large and regular.

Distribution of the classification of nuclei treated with and without HT was performed using the NMA tool and is shown in Figure 2A. A significantly higher percentage of regular small nuclei (SR) was observed for TSL-CDDP and TSL-CDDP-HA hyperthermia groups compared to the groups with no heating, which indicates a higher occurrence of apoptosis by combining CDDP and moderate hyperthermia. However, only the TSL-CDDP showed a significant difference compared to the group treated with the free drug, leading to a higher percentage of LR nuclei. This finding indicates a higher incidence of senescence in the group without hyperthermia treated with TSL-CDDP. Figure 2B shows the fluorescence images of MDA-MB-231 nuclei stained with Hoescht after treatment with the formulations and the free drug, in which the nuclear changes are evident when compared to the control group.

### 3.4. In Vivo Toxicity Study

#### 3.4.1. Assessment of Clinical Signs, Weight and Mortality of Animals

For the in vivo study, we define a CDDP dose of 10 mg/kg to be administered to mice, based on previous studies carried out by our research group [20]. A slight behavioral change was observed for all treated groups, characterized by prostration and mild piloerection within the fourth-hour post-injection. These signs were reverted in the liposomes-treated groups. However, piloerection remained for seven days in two animals from the free CDDP group, in addition to an intestinal imbalance, which resulted in the death of one animal on day 7 (Table 3). On the other hand, no signs of toxicity or death occurred in control (saline), TSL-CDDP and TSL-CDDP-HA treated groups.

**Figure 3:**
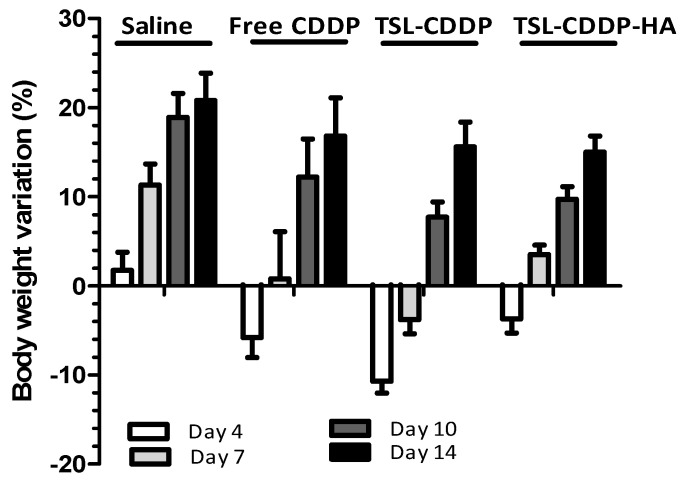
Body weight variation of mice over the 14 days of the experiment. Data were presented as the mean ± standard error of the mean.

The weight variation of the animals over the 14 days of treatment was also evaluated (Figure 3). A gain in body weight was observed in mice treated with saline alone. In contrast, weight loss was observed for all CDDP-treated groups during the first days of analysis. Importantly, a bodyweight recovery was verified from the 7th day for the group treated with TSL-CDDP-HA and from the 10th day for groups treated with free CDDP and TSL-CDDP.

#### 3.4.2. Hematological Investigation

The hematological parameters evaluated are shown in Table 4. There was a significant difference in erythrocyte series parameters for all CDDP-treatments compared to the saline group. For TSL-CDDP, a significant decrease in the erythrocyte series and an increase in the white blood cell count were observed compared to the free CDDP group. On the other hand, no significant difference in hematological parameters between TSL-CDDP-HA and the free drug was observed.

#### 3.4.3. Biochemical Investigation

Table 5 presents the biochemical parameters indicative of renal (urea and creatinine) and hepatic (ALT and AST) toxicity. Renal toxicity caused by the treatments TSL-CDDP and free CDDP was observed, due to the toxic potential already expected for these formulations. On the other hand, the formulation containing HA was able to reduce kidney damage, since no difference was observed compared to the saline group. However, there was a marked increase in liver enzymes (AST and ALT) in the group treated with this formulation.

#### 3.4.4. Histopathological Investigation

**Figure 4:**
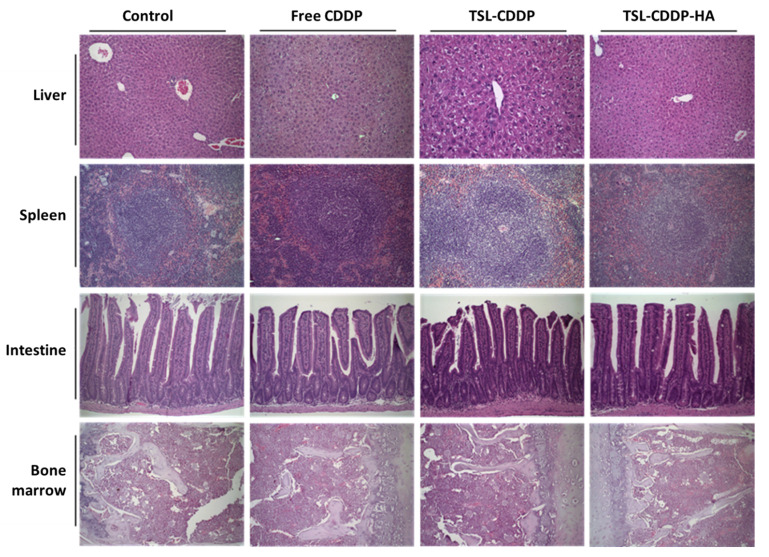
Photomicrographs of liver, spleen, intestine and bone marrow from Swiss mice treated with saline (control), free CDDP, TSL-CDDP and TSL-CDDP-HA. Hematoxylin & eosin staining. 20× magnification.

**Figure 5:**
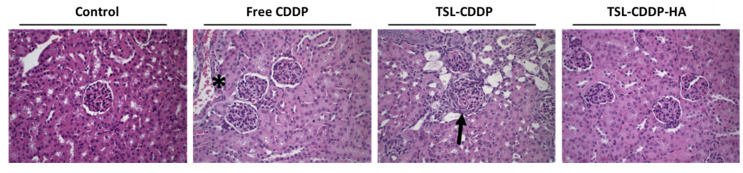
Photomicrographs of kidneys from Swiss mice treated with saline (control), free CDDP, TSL-CDDP and TSL-CDDP-HA. Arrow indicates the thickening of Bowman’s capsule, asterisk indicates inflammatory infiltrate and fibrosis. Hematoxylin & eosin staining. 40× magnification.

Histological analysis of liver, spleen, intestine, kidney and bone marrow was performed for all studied groups (Figure 4 and Figure 5). No apparent alteration was found in any organ in the control group and in the TSL-CDDP-HA treated group.

In the group treated with free CDDP, inflammatory infiltrate and fibrosis were observed in the kidney in only one animal (Figure 5). Renal alterations caused by free CDDP were found in previous studies carried out by our research group only for a dose of 20 mg/kg [20]. For animals treated with TSL-CDDP, a focal glomerular lesion, indicated by a thickening of Bowman’s capsule (Figure 5), was observed in all animals evaluated. These results show a direct correlation between high blood urea levels and the presence of renal alteration, corroborating the high toxicity observed for cationic liposomes. However, this finding was only focal, indicating a possible recovery of the animals. This renal toxicity caused by the cationic formulation was successfully eliminated with the use of the targeting ligand (HA) since the group treated with TSL-CDDP-HA did not present any histological alteration in the analyzed organ.

## 4. Discussion

It is known that CDDP treatment is related to several toxic effects, mainly nephrotoxicity [1]. Nano-systems have been studied in an attempt to minimize these damages; however, conventional liposomes have some limitations such as rapid blood clearance and lack of tumor specificity, among other problems [23]. In this context, the use of surface ligands can be applied in an attempt to improve blood circulation time, in addition to being able to act as a target for the tumor, since the tumor region overexpresses receptors for some ligands, such as HA [13]. Another alternative to improve the effectiveness of liposomes is the use of thermosensitive lipids, capable of triggering drug release only after local (tumor) heating [6]. This new therapeutic alternative may contribute to a reduction or elimination of systemic toxic effects.

In this study, the in vitro activity and in vivo toxicity of a new thermosensitive formulation developed by our research group were evaluated. The proposal was to study this new delivery system to verify its potential antitumor activity and toxicological profile to allow evaluation in an experimental model of breast cancer. Thus, a series of pre-clinical studies were carried out, such as cell viability, IC50, nuclear morphology and in vivo toxicity, which included body weight variation, mortality, hematological and biochemical profiles (renal and hepatic), besides histopathological evaluation after intravenous administration in female Swiss mice.

The in vitro analyses allowed us to observe greater cytotoxicity of the cationic formulation (TSL-CDDP) against the MDA-MB-231 cells compared to the free CDDP (Table 2). This finding suggests that TSL-CDDP favors cellular uptake of the drug, with cells being more susceptible to the action of CDDP. The same was observed by Sun and coworkers in a study with cationic CDDP liposomes, in which increased cytotoxicity was also related to increased cellular uptake [24]. This higher cytotoxicity observed for TSL-CDDP may be related to its positive zeta potential (+20 mV) provided by the presence of SA. The relationship between positive surface charge and interaction with cell membranes has already been reported in the literature, revealing a decrease in cell viability when treated with positively charged liposomes [24,25,26].

Still dealing with in vitro analyses, it is known that several cellular mechanisms affect nuclear morphology and, therefore, can be used to assess drug action mechanisms [22]. The nucleus corresponds to approximately 10% of the cell volume and, due to its nuclear envelope, has a rounded shape and a well-defined and regular surface under normal in vitro conditions. Nuclear condensation and fragmentation are observed in the process of apoptosis and an increase in nuclear size indicates senescence, while irregularity occurs due to chemical or physical stress, or exogenous agents that affect microtubule dynamics [27]. In this analysis, TSL-CDDP and TSL-CDDP-HA treatment with hyperthermia led to cell apoptosis. Cells undergoing apoptosis show a high and regular condensation of their nucleus, which occurs before nuclear fragmentation [28]. Due to this high condensation in an almost spherical shape, it has been hypothesized that the nuclei of cells undergoing apoptosis may appear as small and regular [22]. Furthermore, TSL-CDDP exhibited greater formation of LR nuclei compared to the free CDDP (Figure 2), indicating a higher occurrence of senescence in tumor cells treated with this formulation. Thus, once again it is possible to suggest greater toxicity of TSL-CDDP due to its positive charge. Cancer cell senescence is a state of cell arrest that often occurs in response to therapy. It is often called therapy-induced senescence (TIS) or accelerated cellular senescence to differentiate it from the aging process of normal cells, known as replicative senescence [29]. Milczarek et al. published a review on senescence in breast cancer and highlighted that CDDP triggered senescence in MDA-MB-231 strains and that the expression of 21 genes responsible for TNBC resistance to CDDP is linked to senescence [30].

However, in vivo, these findings were not beneficial since the TSL-CDDP toxicity is not selective for tumor cells, causing damage to healthy cells as well. In this sense, alterations were observed in the hematological exams for the groups treated with TSL-CDDP compared to the free CDDP. The leukocytosis and lymphocytosis observed for this formulation may be related to some inflammatory process resulting from the toxicity caused by the greater interaction of the cationic liposome with blood cells. A similar response was found by Filion and Phillips [25], in which cationic liposomes formulated with SA were shown to interact with serum proteins and red blood cells, inducing a strong coagulation response and hemolysis, and an important laboratory alteration was lymphocytosis [25]. The same was observed in the data from biochemical tests since TSL-CDDP was not able to prevent the kidney damage caused by CDDP (Table 5), which was circumvented by the use of HA on the surface of the liposomes. On the other hand, the use of this ligand led to an increase in hepatic enzymes in plasma, which is related to some injury to the liver, causing the death of hepatocytes that are consequently detected in the blood. This finding is justified by the presence of HA endocytosis receptors, highly expressed in sinusoidal endothelial cells of lymph nodes, liver and spleen [31]. However, data from the histological analysis show that, despite this alteration in the biochemical examination, no histological alteration was detected in the liver of the animals treated with TSL-CDDP-HA. Histological sections at different levels of the hepatic and splenic parenchyma were within normal limits, with the architecture of the organs preserved (Figure 4).

Still, as a consequence of the toxicity due to the positive charge on the surface of TSL-CDDP, greater weight loss was also observed in animals compared to the free CCDP (Figure 3). On the other hand, HA-coated liposome was able to eliminate kidney damage in vivo and showed less weight loss in animals treated with this formulation (Table 5 and Figure 3). It is also worth mentioning that the histological analysis of the other organs of the animals treated with TSL-CDDP-AH did not show any microscopic alteration in the organs evaluated (Figure 4 and Figure 5).

In conclusion, the results suggest that the innovative thermosensitive formulation of CDDP functionalized with HA by electrostatic interaction may represent an advance in the reduction of CDDP-related toxicity, configuring a therapeutic advantage in the treatment of breast cancer, since one of the main problems related to the use of CDDP is drug-induced toxicity.

## Figures and Tables

**Table 1:** Characterization of cisplatin thermosensitive liposomes.

	Formulations	TSL-CDDP	TSL-CDDP-HA
Parameters			
Mean Diameter (nm)		104.1 ± 1.9	103.6 ± 2.2
PDI		0.14 ± 0.01	0.06 ± 0.01
Zeta Potential (mV)		+21.4 ± 0.3	+0.3 ± 1.4 *
Encapsulation (μg/mL)		266.3 ± 19.9	239.7 ± 12.7
EP (%)		13.7 ± 1.8	13.1 ± 0.9

TSL-CDDP: thermosensitive liposome containing CDDP; TSL-CDDP-HA: thermosensitive liposome functionalized with HA (24 mg/mL) by electrostatic interaction. Mean initial cisplatin concentration: 1.8 ± 0.1 mg/mL. Data expressed as mean ± standard error (*n* = 3). * represents significant difference compared to TSL-CDDP (*p* < 0.05, Tukey’s test).

**Table 2:** IC50 and confidence interval in MDA-MB-231 cell lines.

Treatments	Without HT		With HT	
	IC_50_ (µM)	CI	IC_50_ (μM)	CI
Free CDDP	50.3	37.5 to 67.2	42.1	32.2 to 54.9
TSL-CDDP	16.6 *	11.4 to 24.1	17.0	10.9 to 26.5
TSL-CDDP-HA	30.6	21.4 to 43.6	28.3	21.6 to 37.0

Data are expressed as mean and confidence interval (CI) of three independent experiments. * Represents a significant difference when compared to free CDDP (*p* < 0.05, Student *t*-test). Abbreviations: IC50: concentration of CDDP required to promote 50% inhibition of cell viability.

**Table 3:** Mortality of mice over the 14 days of the experiment.

Treatments	Number of Dead Animals/Number of Treaties	Day of Death
Saline	0/6	-
TSL-CDDP	0/6	-
TSL-CDDP-HA	0/6	-
Free CDDP	1/6	7

**Table 4:** Hematological parameters of Swiss mice treated with saline, TSL-CDDP, TSL-CDDP-HA and free CDDP at dose of 10 mg/kg.

Parameters	Saline	Free CDDP	TSL-CDDP	TSL-CDDP-HA
WBC (10^3^/µL)	5.1 ± 0.3	4.7 ± 0.5	7.8 ± 0.9 ^abc^	4.9 ± 0.6
LINF (10^3^/µL)	2.8 ± 0.2	2.8 ± 0.3	5.0 ± 0.6 ^abc^	2.8 ± 0.3
RBC (10^6^/µL)	6.4 ± 0.1	5.6 ± 0.1 ^b^	5.2 ± 0.1 ^abc^	5.7 ± 0.1 ^b^
HGB (g/dL)	13.2 ± 0.2	11.4 ± 0.3 ^b^	10.5 ± 0.2 ^ab^	11.4 ± 0.1 ^b^
HCT (%)	34.1 ± 0.6	31.1 ± 0.9 ^b^	27.7 ± 0.5 ^bc^	30.1 ± 0.5 ^b^
RDW (%)	14.9 ± 0.3	15.4 ± 0.6	16.2 ± 0.1 ^b^	15.5 ± 0.2
PLT (10^3^/µL)	251.5 ± 11.5	233.6 ± 16.3	283.1 ± 28.8	276.7 ± 16.9

Data represented as mean ± standard error of mean (*n* = 6 animals), except for free CDDP (*n* = 5). ^a^ represents significant difference compared to the TSL-CDDP-HA group. ^b^ represents significant difference compared to the saline group. ^c^ represents significant difference compared to the free CDDP group. *p* < 0.05 (Tukey test). Abbreviations: WBC: white blood cell count; LYMP: lymphocytes; RBC: red blood cell count; HGB: hemoglobin; HCT: hematocrit; RDW: distribution of red blood cells; PLT: platelet count.

**Table 5:** Biochemical parameters of Swiss mice treated with saline, TSL-CDDP, TSL-CDDP-HA and free CDDP at dose of 10 mg/kg.

Parameters	Saline	Free CDDP	TSL-CDDP	TSL-CDDP-HA
Urea (mg/dL)	49.2 ± 2.8	66.5 ± 1.0 ^b^	73.1 ± 2.4 ^ab^	59.3 ± 4.6
Creatinine(mg/dL)	0.3 ± 0.01	0.4 ± 0.01 ^b^	0.3 ± 0.01	0.3 ± 0.01 ^c^
AST (U/L)	89.8 ± 6.3	78.1 ± 2.4	94.9 ± 5.2 ^a^	121.1 ± 10.2 ^bc^
ALT (U/L)	37.2 ± 2.1	43.5 ± 3.8	44.9 ± 2.8 ^a^	65.1 ± 7.6 ^bc^

AST: aspartate aminotransferase. ALT: alanine aminotransferase. Data represented as mean ± error of 6 animals, except for free CDDP (*n* = 5). ^a^ represents a significant difference in relation to the TSL-CDDP-HA group. ^b^ represents a significant difference in relation to the saline group. ^c^ represents a significant difference in relation to the free CDDP group. *p* < 0.05 (Tukey test).

## Data Availability

Not applicable.
